# Correlation of Volume of Macular Edema with Retinal Tomography Features in Diabetic Retinopathy Eyes

**DOI:** 10.3390/jpm11121337

**Published:** 2021-12-09

**Authors:** Santosh Gopi Krishna Gadde, Arpita Kshirsagar, Neha Anegondi, Thirumalesh B. Mochi, Stephane Heymans, Arkasubhra Ghosh, Abhijit Sinha Roy

**Affiliations:** 1 Department of Retina, Narayana Nethralaya Eye Hospital, Bangalore 560099, India; drsantoshgk@gmail.com (S.G.K.G.); thirumaleshmb@gmail.com (T.B.M.); 2 Imaging, Biomechanics and Mathematical Modelling Solutions Lab, Narayana Nethralaya Foundation, Bangalore 560099, India; arpitakshirsagar@gmail.com (A.K.); nanegondi@gmail.com (N.A.); 3 Department of Cardiology, CARIM School for Cardiovascular Diseases, Maastricht University, Universiteitssingel 50, 6229 ER Maastricht, The Netherlands; stephane.heymans@mumc.nl; 4 Centre for Molecular and Vascular Biology, Department of Cardiovascular Sciences, KU Leuven, Herestraat 49, bus 911, 3000 Leuven, Belgium; 5 GROW Research Laboratory, Narayana Nethralaya Foundation, Bangalore 560099, India

**Keywords:** diabetic retinopathy, retina, edema, OCT, tomography

## Abstract

Optical coherence tomography (OCT) enables the detection of macular edema, a significant pathological outcome of diabetic retinopathy (DR). The aim of the study was to correlate edema volume with the severity of diabetic retinopathy and response to treatment with intravitreal injections (compared to baseline). Diabetic retinopathy (DR; *n* = 181) eyes were imaged with OCT (Heidelberg Engineering, Germany). They were grouped as responders (a decrease in thickness after intravitreal injection of Bevacizumab), non-responders (persistent edema or reduced decrease in thickness), recurrent (recurrence of edema after injection), and treatment naïve (no change in edema at follow-up without any injection). The post-treatment imaging of eyes was included for all groups, except for the treatment naïve group. All eyes underwent a 9 × 6 mm raster scan to measure the edema volume (EV). Central foveal thickness (CFT), central foveal volume (CFV), and total retinal volume (TRV) were obtained from the early treatment diabetic retinopathy study (ETDRS) map. The median EV increased with DR severity, with PDR having the greatest EV (4.01 mm^3^). This correlated positively with TRV (*p* < 0.001). Median CFV and CFT were the greatest in severe NPDR. Median EV was the greatest in the recurrent eyes (4.675 mm^3^) and lowest (1.6 mm^3^) in the treatment naïve group. Responders and non-responders groups had median values of 3.65 and 3.93 mm^3^, respectively. This trend was not observed with CFV, CFT, and TRV. A linear regression yielded threshold values of CFV (~0.3 mm^3^), CFT (~386 µm), and TRV (~9.06 mm^3^), above which EV may be detected by the current scanner. In this study, EV provided a better distinction between the response groups when compared to retinal tomography parameters. The EV increased with disease severity. Thus, EV can be a more precise parameter to identify subclinical edema and aid in better treatment planning.

## 1. Introduction

Diabetic retinopathy (DR) is the main complication of both type one and type two diabetes and is one of the leading causes of blindness [1]. Diabetic macular edema (DME) results from retinal microvascular changes that compromise the blood-retinal barrier [2]. The earliest signs of leakage have been observed in mild, non-proliferative DR (NPDR), and increase with higher grades of DR (moderate and severe), until the end stage, where it reaches the proliferative stage (PDR) [3]. The development of optical coherence tomography (OCT) has allowed the imaging of DME with high resolution tomography. Since the classification of DR is primarily based on fundus image features^3^, there is a clinical need of the improved quantification of DME. Several studies in the recent past have attempted the quantification of DME zones in 2-D OCT images. These studies can be broadly classified into two types: (a) studies where the clinical evaluation of DME led to improved understanding of the disease and response to therapy [4,5,6]; (b) studies where methods were developed to automatically detect DME and to classify the disease accordingly [7,8,9,10]. However, none of these studies quantified the morphological featured of edema in terms of 3D tomography, i.e., the volume of the edema after treatment, and its correlation with other clinical imaging features. Therefore, this study analyzed the distribution of edema volume in a population of DME patients, who were either being treated to resolve their edema or were yet to be treated. Subsequently, the volume of their edema was correlated with current clinical imaging features, namely, central foveal volume (CFV), central foveal thickness (CFT), and total retinal volume (TRV), to establish the proportionate change in the volume of an edema for a specific change in a clinical feature after treatment. These clinical features were derived from early treatment diabetic retinopathy sectors (ETDRS) [11].

## 2. Methods

This was a retrospective, observational, cross-sectional study of the eyes of diabetic patients who were diagnosed with retinopathy. The study was approved by the Narayana Nethralaya institutional Ethics Committee, Bangalore, India. All methods were performed in accordance with the relevant guidelines and regulations of the hospital, as set by the Ethics Committee. Medical records of the patients were retrospectively reviewed, and the need for patient consent was waived by the Ethics Committee. Only those eyes having had B-scans, which were acquired with the same scan protocol, were chosen for retrospective analyses. The sample size was 181 patients, in the age range from 38 to 79 years. Among these, 19 patients did not have edema. The remaining patients (n = 162) were sub-divided into the following response sub-groups:

(a) Responders—decrease in CFT by 100 μm or more after either the first or second intravitreal injection of steroid or anti-VEGF; (n = 60)
(b) Non-responders—either persisting macular edema or having an increase in CFT by 100 µm or CFT ≤ 100 µm from previous OCT scans, after 3 consecutive intravitreal injections; (n = 63)
(c) Recurrent—return of macular edema with an increase in thickness greater than 100 μm, when compared to the last visit, after an injection free period of 2 months; (n = 26)
(d) Treatment naïve—no visible signs of change in macular edema from consecutive scans at regular follow-ups, or eyes without any prescribed treatment. (n = 29)

In responder, non-responder, and recurrent eyes, the post-treatment scans were analyzed. In the treatment naïve, there was no treatment involved by definition.

In case of anti-VEGF injections, patients other than the treatment naïve group had undergone intravitreous injections of bevacizumab (Roche, Basel, Switzerland). Recent studies have suggested the classification of responders and non-responders based on more than three repeat intravitreous injections [12,13]. In the Indian sub-continent, treatment with repeat injections is limited, due to the high costs of the injections. Thus, it becomes necessary to evaluate the treatment outcomes earlier than the protocols followed in Western populations. We aimed to investigate the distribution of tomographic and edema volume in the aforementioned subgroups after intravitreous injection and compare them with treatment naïve patients. Furthermore, only those patients with an identical central macular raster scan pattern, and without significant media haze based on visual examination by retina specialists (S.G, T.M), were included in the study.

Exclusion criteria included the presence of vitreous hemorrhage, coexistent uveitis, having had ocular surgery in the last 6 weeks, any intravitreal injections for other causes, presence of vein occlusions, age related macular degeneration, pseudophakic cystoid macular edema, and endophthalmitis. All the OCT scans were performed with a Heidelberg Spectralis™ (Heidelberg Engineering, Heidelberg, Germany). This device had an A-scan rate of 40,000 lines per second. The images were exported as zipped files (*.e2e format) and converted to video files (*.avi) using the Heidelberg Eye. The frames in the video files were extracted as images (*.png) for postprocessing. CFV and CFT were obtained from the central sector of the ETDRS thickness map. TRV was obtained by adding the volumes from all the sectors of the ETDRS map.

To calculate the volume of the edema, a 9 × 6 mm scan of the central retina, centered approximately at the fovea, was acquired. A total of 25 uniformly spaced B-scans were acquired within the scan area. Only those scans were used, where all the 25 B-scans had a maximum noise level of 90 or below. The B-scans were exported from the OCT device as 8-bit gray scale images for analyses. Each scan was further processed to quantify the edema zones. Since the edema was 3D in shape, each B-scan was essentially a 2D cross-sectional image of a 3D volume. Each B-scan was initially resolved with a Wiener filter (window size 5 × 5), which preserved the high frequency sections of the B-scan. Since the amount of noise can vary among the images, as well as signal strength, signal to noise ratio (SNR) balancing was used to further resolve the edema zones. The evident noise *N* in the image was computed as the mean pixel value within a window in the upper left portion of the image. The signal *S* was calculated as the mean pixel value within a window located from the rightmost image, where the signal value was high. The noise and signal values were chosen after a trial and error method, wherein images with different signal strength and noise values were considered. The values for *N* and *S* were averaged across the B scan images. The images were then SNR balanced using the equation

*I_f_* = (*I*_0_ − *N*)/(*S* − *N*)

where *I*_0_ is the initial pixel value and *I_f_* is the final pixel value [14]. This equation was applied to each B scan. Then, a median filter was applied to these SNR balanced images (window size of 15 × 15) to remove salt and pepper noise. A morphological operation was performed to isolate the edema zones with a minimum number of connected components [15]. The anterior and posterior boundary of the retina was segmented in each B-scan to restrict the quantification only to the region of interest (ROI). The anterior boundary (inner limiting membrane) was defined as the layer having the first horizontal gradient change from the top. Similarly, the posterior boundary (retinal pigment epithelium) was defined as the first gradient change in the horizontal direction from the bottom. All the dark pixels (intensity~0) in the ROI were identified as edema. The segmented edema regions were overlaid on the original image. The area occupied by the pixels was calculated and converted to millimeters squared (image pixel resolution was ~3.77 pixel/mm).

In this study, all or some of the 2D B-scans for a given eye captured the cross-sections of edema regions, if they existed at the location of the scan. Then, the area of the captured cross-section of the edema was calculated from the image. These areas were calculated for all 25 of the B-scans. Since the spatial separation between the B-scans was known (25 µm), the areas were simply integrated numerically using the Trapezoidal method of integration. If A_i_ represented the area of the edema region in the i^th^ B-scan, then the edema volume was computed as:

$$\mathrm{Volume}\mathrm{of}\mathrm{edema}=\left(h/2.0\right)\times \left(2\sum_{i=2}^{24}A_{i}+A_{1}+A_{25}\right)$$

where h = 25 µm. This provided an estimate of the total volume of the edema within the scan area of 9 × 6 mm. Since volume is essentially the sum of all the areas, the difference in volume computed by areas that were derived from manual segmentation vs. an algorithm would be simply the sum of differences between the individual areas derived from the manual vs. algorithm segmentation of the edema cross-sections. All the above methods were implemented using MATLAB v7.10 (MathWorks Inc., Natick, MA, USA).

### Statistical Analyses

The normality of distribution was checked with the Kolmogorov-Smirnov test. All continuous variables were reported as median along with 95% of CI interval. Additionally, a manual segmentation of the edema zones was performed by an experienced retina specialist for a sub-set of the total number of B-scans. The agreement between the manual and automated segmentation of the area of the edema zones in the B-scans was analyzed with ICC. This agreement between the areas of fluid-filled zones in the B-scans was also equal to the agreement between corresponding volumes of the fluid filled zones. The variables analyzed were the corrected distance visual acuity (CDVA in LogMAR), volume of the edema (mm^3^), CFV (mm^3^), CFT (μm), and TRV (mm^3^). The variables were analyzed between the ETDRS grades and also between the response sub-groups.

The Kruskal Wallis test was used for the pairwise comparison of subgroups, using the Conover post hoc test. A *p*-value of less than 0.05 was considered statistically significant. The statistical analyses were performed with MedCalc v18.5 (MedCalc Inc., Ostend, Belgium).

## 3. Results

### 3.1. Segmentation and Edema Volume Calculation

The manual segmentation of edema zones was performed in 371 B-scans chosen randomly from the patient scans. The areas of segmented zones were calculated from both the manual method and from the algorithm. The intra-class correlation (ICC) between the manual and automated segmentation of areas was 0.91 (95% confidence interval ADDIN EN.CITE [1] 0.89–0.93). Among the 181 eyes, 19 were devoid of edema and were isolated as a separate group. The CFV, CFT, and TRV of these eyes were 0.31 (0.28–0.34) mm^3^, 395 (342.4–439.2) μm and 10.1 (9.24–12.20) mm^3^, respectively. Among the eyes with edema, there were 7 with mild NPDR, 44 with moderate NPDR, 53 with severe NPDR and 74 with PDR.

### 3.2. Tomographic Features vs. DR Grades

Table 1 summarizes the salient tomographic features of the DR eyes stratified on the basis of severity. Corrected distance visual acuity (CDVA), TRV, CFV, and CFT were similar between the grades of DR eyes (*p* > 0.05). However, edema volume increased with an increasing severity of the grade of DR (*p* < 0.001). Among the response groups, there were 60 responder eyes, 63 non-responder eyes, 26 recurrent eyes, and 29 treatment naïve eyes.

### 3.3. Tomographic Features vs. Treatment Response

Table 2 summarizes the salient tomographic features of the DR eyes stratified on the basis of response to treatment. Only CDVA was better in the responder and treatment naïve groups when compared to the non-responder and recurrent groups (*p* = 0.05). The remaining indices were similar between the groups (*p* > 0.05). The volume of edema was greatest in the recurrent group (median of 4.675 mm^3^). The linear correlation between CDVA and the edema volume was not significant (*p* > 0.05), irrespective of either grade of DR or response to treatment.

### 3.4. Correlation among Features

**Figure 1:**
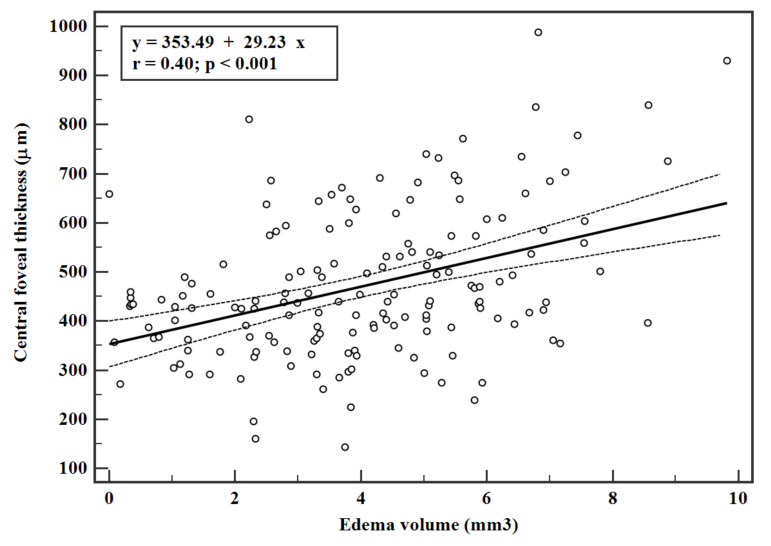
Linear regression with 95% confidence interval between central foveal thickness and edema volume. Only eyes with an edema volume greater than zero were included in the regression. Thickness was derived from the central sector of the ETDRS map.

**Figure 2:**
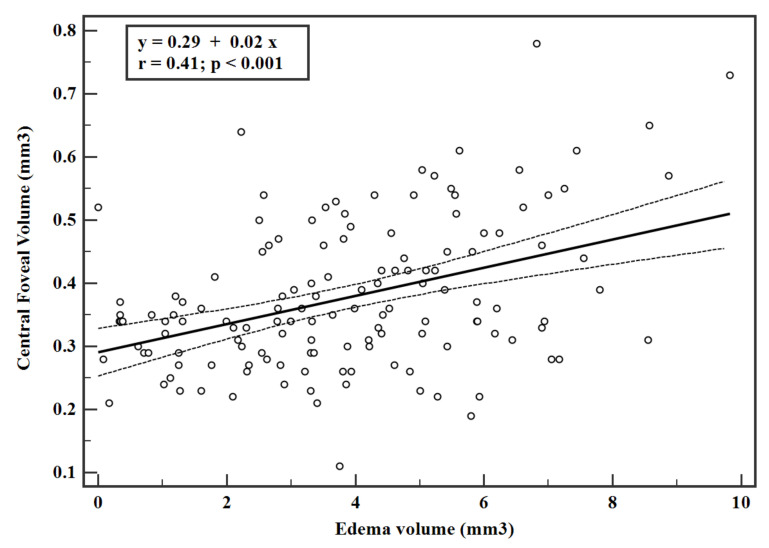
Linear regression with 95% confidence interval between central foveal volume and edema volume. Only eyes with an edema volume greater than zero were included in the regression. Volume was derived from the central sector of the ETDRS map.

**Figure 3:**
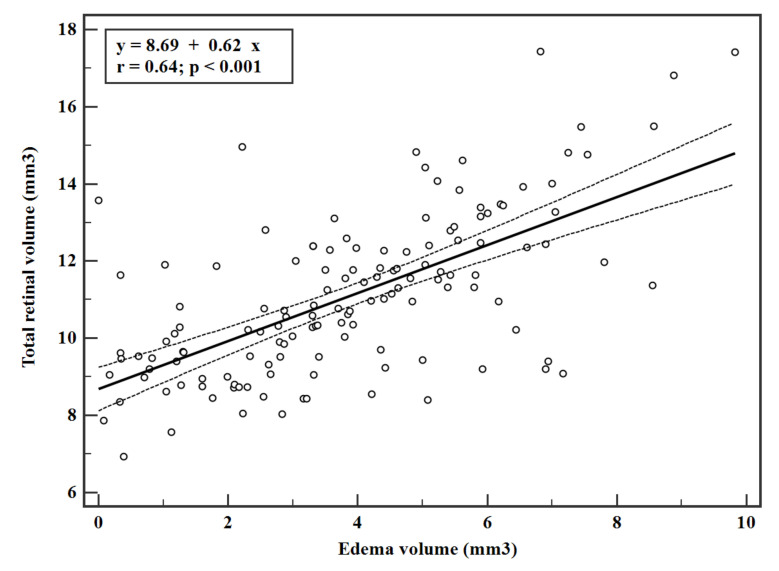
Linear regression with 95% confidence interval between total retinal volume and edema volume. Only eyes with an edema volume greater than zero were included in the regression. Total retinal volume was derived by adding the volumes of all the sectors of the ETDRS map.

**Figure 4:**
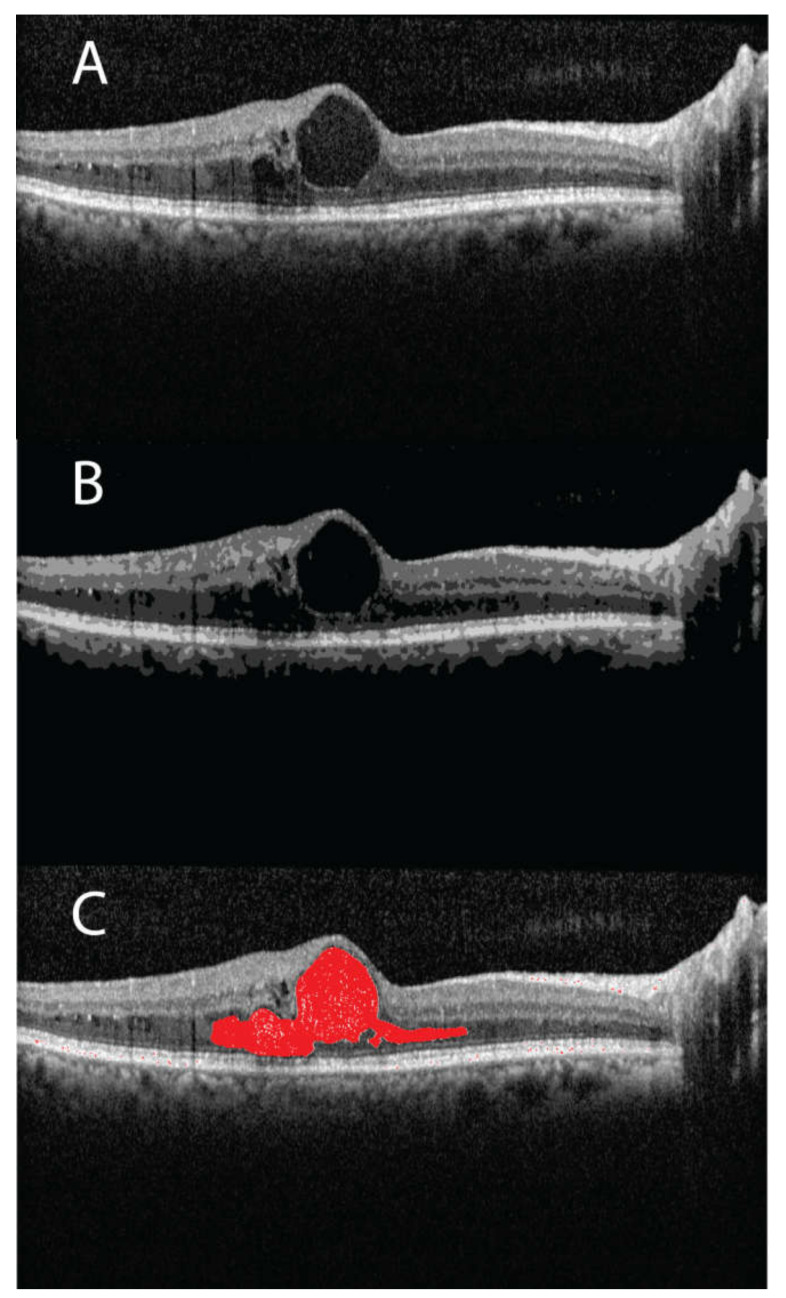
(**A**) A sample B-scan of a patient (categorized as recurrent and with an ETDRS grading as PDR) showing fluid filled regions; (**B**) the same B-scan is shown after signal to noise ratio balancing; (**C**) segmented fluid region is highlighted in red.

The CFT and edema volume were significantly correlated (r = 0.40, *p* < 0.001) as shown in the linear regression data (Figure 1). Similarly, the linear regression data of CFV and TRV were significantly correlated with the edema volume (r = 0.41 and 0.65 respectively, *p* < 0.001 (Figure 2 and Figure 3)). Interestingly, the estimated zero edema volume CFT (Figure 1) was 386.06 μm, which was very close to the median CFT of the 19 eyes with an absence of edema. Similarly, the zero edema volume CFV (Figure 2) was 0.30 mm^3^, which was very close to the median CFV of the same 19 eyes. In case of TRV, the difference between the two was slightly greater (9.06 mm^3^ from Figure 3 versus 10.1 mm^3^ in the 19 eyes). Figure 4 shows a sample B-scan (A), after SNR balancing, (B) with the segmented edema regions overlaid on (A).

## 4. Discussion

The identification and quantification of edema in DR eyes is a subject of immense interest. With the advent of OCT, the high resolution mapping of edema zones is possible, along with the segmentation of the retinal layers. Increases in retinal thickness due to edema is a primary endpoint in the assessment of edema in DR eyes [4,5,6,16]. The same can be used to assess the efficacy of treatments and progression of the disease as well. Recent studies have focused on the automated classification of DR, by detection of edema in diabetic patients [7,8,9]. However, none of these studies assessed the volume of the edema as a primary endpoint. In this study, the volume of the edema was quantified using serial B-scans and numerical integration to obtain a representative measure of the fluid volume. These were then compared with the clinical classification of DR and its response to treatment, to assess whether current classifications accurately capture change in edema volume, if any.

The calculation of edema volume over a large scan is an important feature of the retina in DR, since the edema may not be localized in the foveal region alone and the region of maximal edema could be away from the fovea. Thus, quantification of the volume of the edema over a larger scan area makes clinical sense. In this study, CFV and TRV were derived from the ETDRS maps with diameters of 1 mm and 3 mm, respectively. In other words, TRV was calculated for an *en face* area of 28.3 mm^2^ (=π × 3 × 3) using 12 radial scans. However, the edema volume was calculated for an *en face* area of 54 mm^2^ (=9 × 6) using 25 rectangular scans. Despite this difference, the linear correlations predicted nearly the same CFT, CFV, and TRV at zero edema volume as the 19 eyes with an absence of edema. An absence of edema can also be seen as the absence of large pockets of edema, which are clearly visible to the naked eye. It may also be treated as “dispersed” edema. Therefore, it appeared that progression of DR led to swelling of the retinal tissues before fluid-filled spaces actually formed in the retina. Detection of fluid-filled volume is also limited by the axial and lateral resolution of the OCT scanner, as volumes which are smaller than the resolution cannot be detected. Thus, it may be concluded that a threshold value of CFT~386 µm, CFV~0.3 mm^3^, and TRV~9.06 mm^3^ (Figure 1, Figure 2 and Figure 3) needs to be reached before edema could be detectable by the OCT scanner that was used in this study. These observations need further validation with larger sample sizes and also with different populations. The linear equations may also be used estimate edema volume from known measurements of CFT, CFV, and TRV from ETDRS maps, thereby serving as predictive models.

Past studies have established that the volume of edema should increase with an increasing severity of DR [3,17]. PDR had the greatest edema volume. However, the ETDRS grading scale does not consider the presence or absence of edema in the retina. Thus, several analyzed eyes in the study had no edema. A separate grading scale, based on the tomography of the retina and edema volume, may be useful to further monitor the progression of disease and response to treatment. Among the response groups, the edema volume was the greatest in the non-responders when compared to baseline, followed by the responders and recurrent eyes. Treatment naïve eyes had the least edema volume. These trends make logical sense but weren’t quantified previously. Further, no clear trends were observed with TRV, CFV, and CFT between the response groups. This implies that the quantification of edema volume may be a better marker to assess response to treatment than CFT, CFV, and TRV. These observations also require further validation.

Deep learning was used to quantify the correlation of retinal thickness and fluid volumes with post-treatment CDVA in a relatively large sample size of eyes (n = 629) [18]. Voxel imaging was used in this study [18]. The agreement between the model prediction and the actual CDVA was moderate (R^2^ = 0.21) [18], but better than the correlation between edema volume and CDVA from our study. Another study [19] used deep learning to quantify the macular fluids in AMD, DME, and RVO eyes. Here, volumetric data from Spectralis were used to detect intraretinal cystoid fluid and sub-retinal fluid (SRF), with an AUC of 0.97 and 0.87, respectively. One more study [20] used deep learning to associate anti-VEGF treatment with visual acuity (BCVA), intra-retinal fluid (IRF) and SRF. It was concluded that presence of SRF was associated with low baseline BCVA. Thus, future combinations of deep learning, a larger sample size, and our method of edema volume analysis may improve the correlations presented in this study. The type of treatment and the rate of change of fluid volume during treatment are also important determinants of residual fluid volume in a patient. Combining this with deep learning may further improve the prediction of visual acuity and/or volume of fluid at the end of the treatment. In another study, edema volume was quantified in age-related macular degeneration eyes using volumetric imaging (voxel size of 30 × 30 × 1.95 µm^3^) [21]. The measured fluid volumes ranged from 0 to 2 mm^3^ [21]. Another study used a voxel size of 11.7 × 46.9 × 2.7 µm to image 10 patients, who underwent anti-VEGF injections over a year [20]. Detected volumes decreased over time and ranged from 0 to 1.8 mm^3^ [22]. Thus, volumetric imaging was the primary protocol of interest in earlier studies. Voxel imaging was generally performed over a smaller *en face* area of 6 × 6 mm and took a longer time to acquire [22,23]. In comparison, our method is relatively faster and covered a larger *en face* area of 9 × 6 mm. In the future, agreement between volume quantification from voxel imaging and our method needs to be assessed for the same *en face* area.

A limitation of this study was that different types of edemas were not analyzed separately, and this needs to be addressed in future studies. A study on the correlation of BCVA with inner and outer retinal thickness found that inner retinal thickness negatively correlated with visual acuity, while outer retinal thickness was positively correlated, or had visual acuity gain, in response to the intravitreal injection of ranibizumab [18]. They speculated that the recovery of the outer retina in response to treatment. In future studies, assessing differential correlations between thickness changes in the inner and outer retinal layers, with changes in edema volume following treatment, would be of more value. However, it is clear that the spatial distribution of edema volume was largely heterogeneous over the scan area, since it correlated with TRV but not with CFT and CFV. Both CFT and CFV were specific to the center retina, i.e., the 1 mm diameter circle of the ETDRS map. Since this is a single center, single population study, it is a limitation which we will address in future studies with diverse patient populations in larger cohorts to validate our observations. In summary, the volume of edema was quantified in response groups with DR and was observed to be significantly correlated with retinal tomography features along with disease severity. It was also observed to be an improved differentiator between the response groups.

## Figures and Tables

**Table 1:** Median with range of indices for the ETDRS grades of diabetic retinopathy.

	Mild NPDR	Mod NPDR	Severe NPDR	PDR	*p*-Value
Age (years)	66 (61.4 to 64.8)	64 (64.00 to 66.00)	62 (61.00 to 65.76)	62.5 (57.33 to 65.00)	0.5
CDVA (LogMAR)	0.3 (0.36 to 0.52)	0.18 (0.16 to 0.30)	0.3 (0.18 to 0.48)	0.477 (0.33 to 0.48)	0.02
Edema Volume (mm^3^)	2.86 (0.47 to 3.84)	2.60 (2.17 to 3.69)	3.85 (3.34 to 4.70)	4.011 (3.31 to 4.76)	0.17
Total Retinal Volume (mm^3^)	9.05 (8.40 to 9.79)	9.52 (9.0 to 10.15)	11.82 (10.63 to 12.4)	11.16 (10.75 to 11.80)	<0.001
Central Foveal Volume (mm^3^)	0.33 (0.28 to 0.35)	0.34 (0.32 to 0.38)	0.42 (0.31 to 0.48)	0.34 (0.31 to 0.36)	0.42
Central Foveal Thickness (µm)	416 (359.24 to 443.11)	434.5 (402.8 to 471.93)	461 (400.03 to 536.78)	435 (398.27 to 452.04)	0.58

CDVA—Corrected distance visual acuity, NPDR—Non-proliferative diabetic retinopathy, PDR—Proliferative diabetic retinopathy.

**Table 2:** Median with range of indices for the DR eyes based on response to treatment.

	Non-Responder	Recurrent	Responder	Treatment Naïve	*p*-Value
Age (years)	64 (61.00 to 65.24)	65 (61.00 to 69.05)	64 (59.46 to 66.00)	62 (55.00 to 66.00)	0.56
CDVA (LogMAR)	0.48 (0.42 to 0.60)	0.6 (0.18 to 1.09)	0.3 (0.18 to 0.48)	0.477 (0.18 to 0.48)	0.05
Edema Volume (mm^3^)	3.93 (3.31 to 4.49)	4.675 (2.83 to 5.41)	3.65 (2.69 to 3.96)	1.604 (0.37 to 3.82)	0.11
Total Retinal Volume (mm^3^)	10.59 (9.82 to 11.39)	11.64 (10.81 to 12.42)	10.74 (10.28 to 11.78)	10.23 9 (32 to 11.94)	0.47
Central Foveal Volume (mm^3^)	0.34 (0.32 to 0.36)	0.395 (0.35 to 0.46)	0.33 (0.280 to 0.40)	0.34 (0.31 to 0.37)	0.11
Central Foveal Thickness (µm)	431 (401.69 to 459.80)	494.5 (439.55 to 577.51)	415 (364.07 to 470.43)	438 (397.51 to 457.57)	0.18

## Data Availability

Data is not available.
